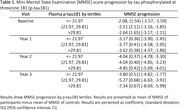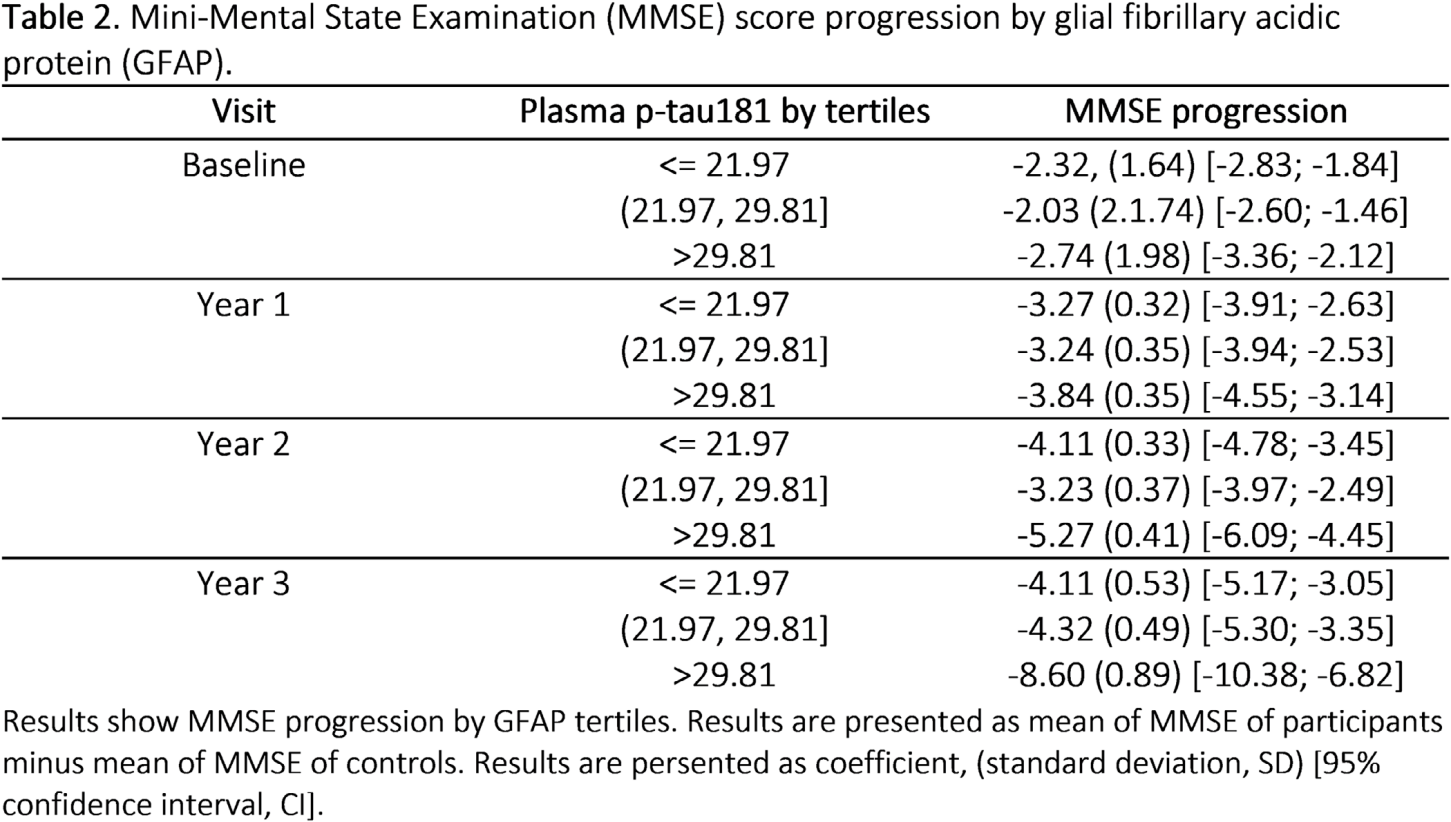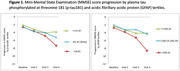# Plasma Biomarkers Predict Cognitive Decline in Alzheimer's Disease

**DOI:** 10.1002/alz.091188

**Published:** 2025-01-09

**Authors:** Núria Guillén, Jordi Sarto, Diana Esteller, Iria Perales, Jose Ríos‐Guillermo, Josep Maria Augé, Laura Naranjo, Raquel Ruiz‐García, Jordi Juncà‐Parella, Adrià Tort‐Merino, Guadalupe Fernandez‐Villullas, Ainoa Alberique, Yolanda González, Anna Antonell, Sergi Borrego‐Écija, Magdalena Castellví, Bea Bosch, Andrea Val‐Guardiola, Mircea Balasa, Raquel Sánchez‐Valle, Neus Falgàs Martínez, Albert Lladó

**Affiliations:** ^1^ Alzheimer’s disease and other cognitive disorders Unit. Hospital Clínic de Barcelona; FRCB‐IDIBAPS; University of Barcelona, Barcelona Spain; ^2^ Medical Statistics Core Facility. IDIBAPS, Hospital Clinic Barcelona. Biostatistics Unit. Faculty of Medicine, Universidad Autónoma de Barcelona, Barcelona Spain; ^3^ Biochemistry and Molecular Genetics Department, Hospital Clínic de Barcelona, Barcelona Spain; ^4^ Immunology Service, Biomedical Diagnostic Center, Hospital Clínic de Barcelona, Barcelona Spain; ^5^ Alzheimer’s disease and other cognitive disorders Unit. Hospital Clínic de Barcelona. Fundació de Recerca Clínic Barcelona – IDIBAPS. University of Barcelona, Barcelona Spain; ^6^ Hospital Clínic de Barcelona ‐ Fundació de Recerca Clínic Barcelona – IDIBAPS ‐ University of Barcelona, Barcelona, Catalonia Spain

## Abstract

**Background:**

Alzheimer's disease (AD) features a complex interplay of factors influencing cognitive decline. While CSF and plasma biomarkers have widely demonstrated their diagnostic utility, whether they may add prognostic value remains unrevealed. With this longitudinal study we aim to address this knowledge gap by evaluating the predictive value of several fluid biomarkers over cognitive decline in a cohort of biomarker‐confirmed AD individuals.

**Method:**

We included 139 participants with biologically‐confirmed AD (A+T+N+). Four cerebrospinal fluid (CSF) biomarkers (Amyloid‐Beta_1‐42_ [Aβ_1‐42_], tau phosphorylated at threonine 181 [p‐tau181], total tau [t‐tau], and neurofilament light chain [NfL]) were determined with enzyme immunoassay, and three plasma biomarkers (p‐tau181, NfL and glial fibrillary acidic protein [GFAP]) were determined with single‐molecule array. Biomarkers were stratified into tertiles. Comprehensive neuropsychological assessments were administered at baseline (n=139) and annually (Year 1 n=108, Year 2 n=78, Year 3 n=25, Year 4 n=3 and Year 5 n=3; mean follow‐up time 1.7 years [SD 0.3]). Mixed Models for Repeated Measures explored the effectof CSF and blood biomarkers on Mini‐Mental State Examination (MMSE) score progression.

**Result:**

Participants had a mean age at onset of 65.7 (SD 6.4) years, 17% were non‐amnestic, 58% were APOEε4 carriers. Higher baseline plasma p‐tau181 and GFAP concentrations correlated with MMSE score decline (p=0.009 and p=0.002, respectively) (Table 1, Table 2, Figure 1). Conversely, no significant associations were observed between plasma NfL or CSF biomarkers concentrations and MMSE decline.

**Conclusion:**

This longitudinal study highlights the potential prognostic value of baseline plasma p‐tau181 and GFAP concentrations for cognitive decline progression in AD.